# Supplementary material for: A smart adhesive Janus hydrogel for non-invasive cardiac repair and tissue adhesion prevention
Source: Nat Commun. 2022 Dec 12;13:7666. doi: 10.1038/s41467-022-35437-5 (PMC9744843; doi:10.1038/s41467-022-35437-5)
Supplement: Supplementary file 1 — Supplementary Information [file 41467_2022_35437_MOESM1_ESM.pdf]

Supplementary Information for

A Smart Adhesive Janus Hydrogel for Non-Invasive cardiac repair and Tissue adhesion prevention

Yutong He[1,2#], Qian Li[2,3], Pinger Chen[2,3], Qixiang Duan[2,3], Jiamian Zhan[2,3], Xiaohui Cai[4], Leyu Wang[2,5], Honghao Hou[2,3]*, Xiaozhong Qiu[1,2]*

1. The Fifth Affiliated Hospital, Southern Medical University, Guangzhou, Guangdong 510900, P. R. China.

2. Guangdong Provincial Key Laboratory of Construction and Detection in Tissue Engineering, Guangzhou, Guangdong 510515, P. R. China.

3. School of Basic Medical Science, Southern Medical University, Guangzhou, Guangdong 510515, P. R. China.

4. School of Pharmaceutical Science, Southern Medical University, Guangzhou, Guangdong 510515, P. R. China.

5. Biomaterials Research Center, School of Biomedical Engineering, Southern Medical University, Guangzhou, Guangdong 510515, P. R. China.

*Corresponding author' Email: ss.hhh89@hotmail.com (H.H.); qqiuxzh@163.com (X.Z.)

These authors jointly supervised this work: Honghao Hou, Xiaozhong Qiu

This file includes: Supplementary Table 1; Supplementary Fig. 1 to 14.

Supplementary Figures and Supplementary Tables

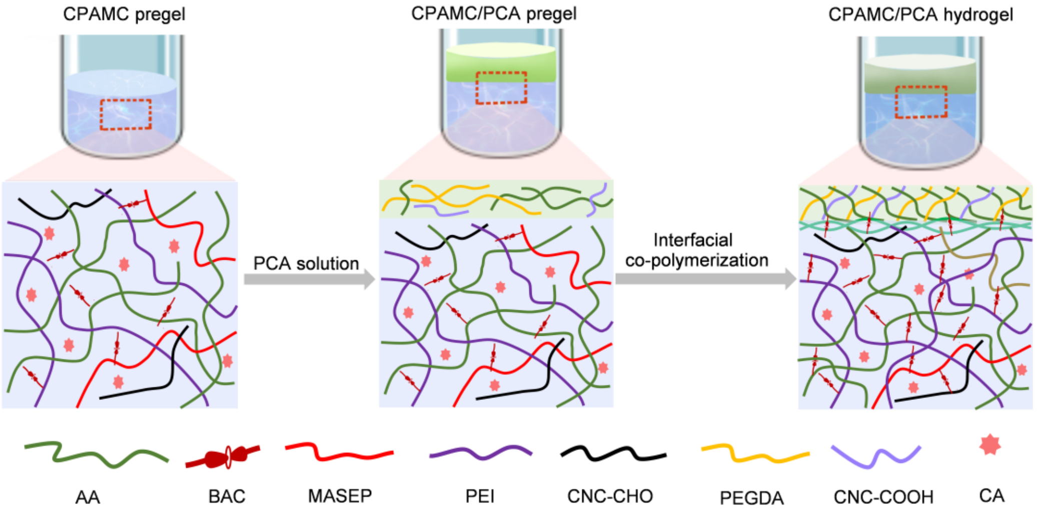

Supplementary Fig. 1. Schematic illustration for the fabrication of CPAMC/PCA Janus hydrogel. AA, acrylic acid; BAC, N, N'-bis(acryloyl) cystamine; MASEP, 3-sulfonic acid propyl methyl acrylic acid potassium; PEI, polyethylenimine; CNC-CHO, aldehyde cellulose; BAC, PEGDA, polyethylene glycol diacrylate; CNC-COOH, carboxylated cellulose; CA, caffeic acid.

Supplementary Table 1. The chemical compositions of various hydrogels.

Hydrogel / Ingredient	CPA	PCA	CPAM	CPAMC	CPAMC/PCA
CNC-CHO	1.25%	-	1.25%	1.25%	1.25%
PEI	1.25%	-	1.25%	1.25%	1.25%
AA	15%	15%	15%	15%	15%
MASEP	-	-	0.2g/mL	0.2g/mL	0.2g/mL
CA	-	-	-	1mg/mL	1mg/mL
BAC	5mg/mL	-	5mg/mL	5mg/mL	5mg/mL
CNC-COOH	-	1%	-	-	1%
PEGDA	-	2%	-	-	2%

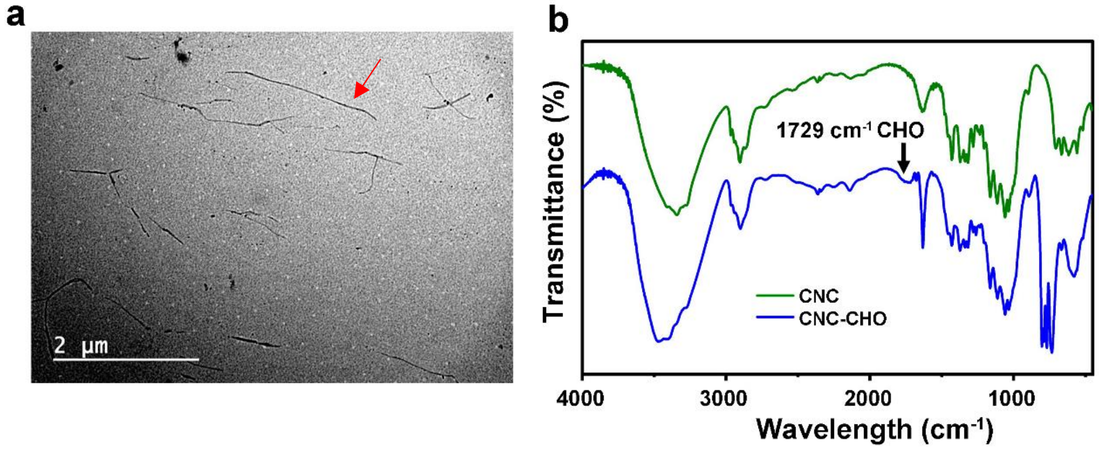

Supplementary Fig. 2. a) The TEM of CNC from sea squirts. b) The FT-IR results of CNC and CNC-CHO. CNC: cellulose naonocrystals

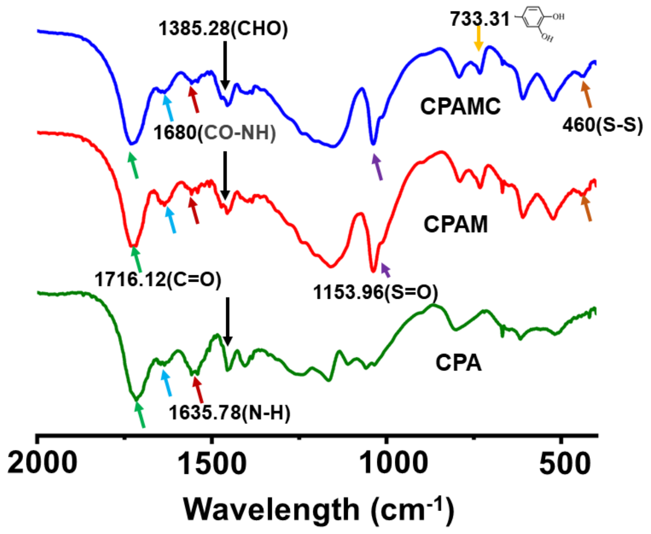

Supplementary Fig. 3. The FT-IR spectra of CPA, CPAM and CPAMC hydrogels. CPA, CPAM, CPAMC: C, aldehyde cellulose (CNC-CHO); P, polyethylenimine (PEI); A, acrylic acid; M, 3-sulfonic acid propyl methyl acrylic acid potassium (MASEP); C, caffeic acid (CA).

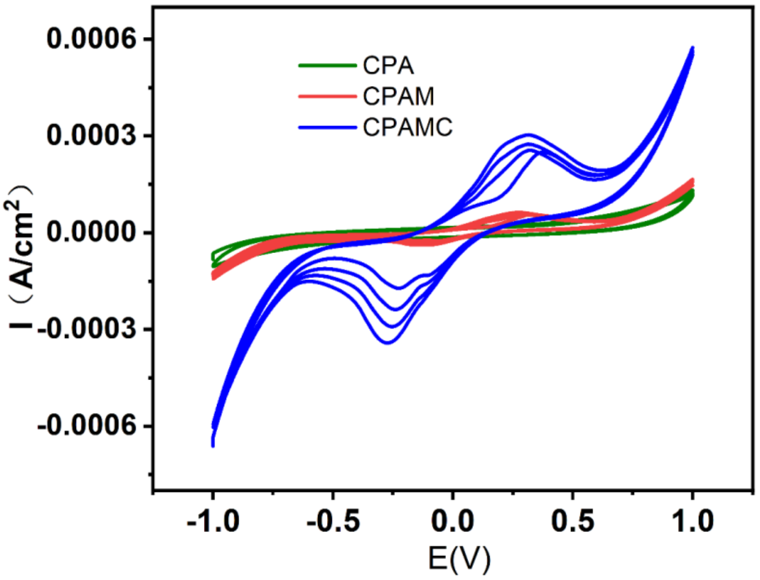

Supplementary Fig. 4. The cyclic voltammetry curves of CPA, CPAM and CPAMC hydrogels. CPA, CPAM, CPAMC: C, aldehyde cellulose (CNC-CHO); P, polyethylenimine (PEI); A, acrylic acid; M, 3-sulfonic acid propyl methyl acrylic acid potassium (MASEP); C, caffeic acid (CA).

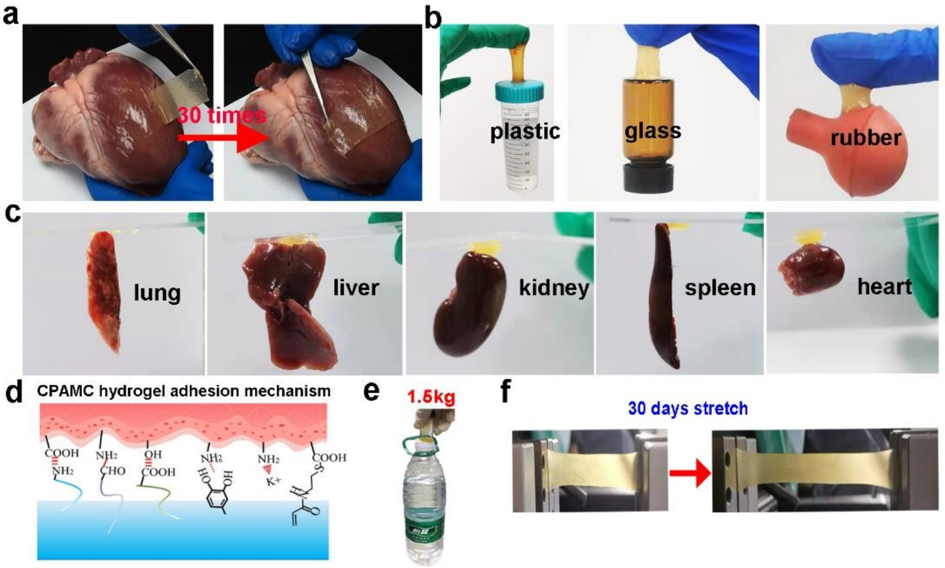

Supplementary Fig. 5. Adhesion performance of CPAMC hydrogel on various tissue organs and materials. a) The CPAMC hydrogel was repeatedly adhered on the heart of the pig over 30 cycles. b-c) The hydrogel was adhered to various material surfaces (b) and tissue organs (c). d) Adhesion mechanism of CPAMC hydrogel to myocardial tissue. e) Adhesion digital photographs of the CPAMC hydrogel supporting a weight

of 1.5 kg. f) Digital photographs showing the good stretching properties of the same CPAMC hydrogel after 30 days of adhesion. CPAMC: C, aldehyde cellulose (CNC-CHO); P, polyethylenimine (PEI); A, acrylic acid; M, 3-sulfonic acid propyl methyl acrylic acid potassium (MASEP); C, caffeic acid (CA).

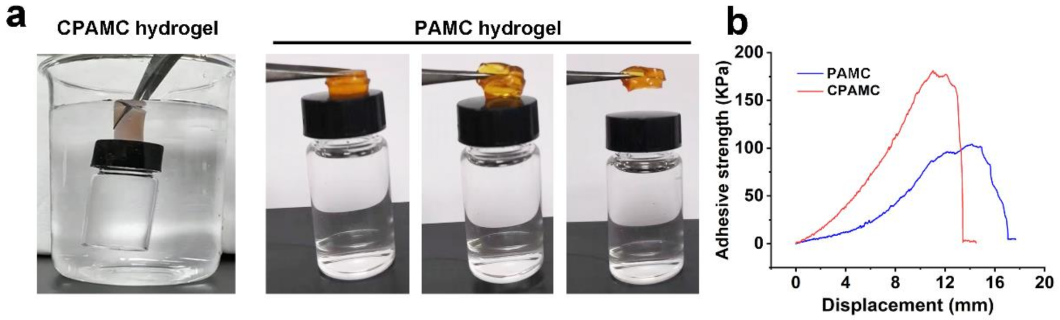

Supplementary Fig. 6. a) Adhesive image of CPAMC hydrogel and PAMC hydrogel. b) Adhesion mechanical curves of CPAMC hydrogels and PAMC hydrogel to glass slide. PAMC, CPAMC: C, aldehyde cellulose (CNC-CHO); P, polyethylenimine (PEI); A, acrylic acid; M, 3-sulfonic acid propyl methyl acrylic acid potassium (MASEP); C, caffeic acid (CA).

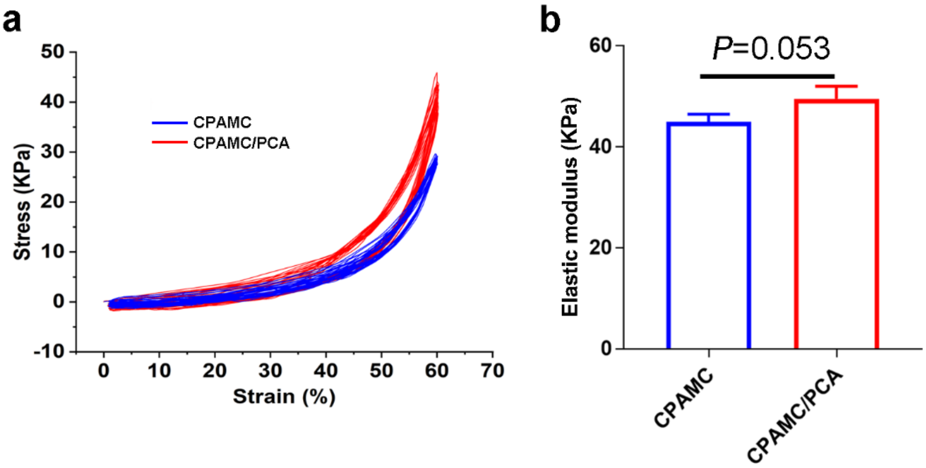

Supplementary Fig. 7. a) The stress-strain curves of CPAMC hydrogel and CPAMC/PCA hydrogel. b) The elastic modulus of CPAMC hydrogel and CPAMC/PCA hydrogel, n=3 independent samples. (Error bar means the standard deviation, *p < 0.05, **p < 0.01, ***p < 0.001, p value was generated by t test, n=

independent samples). CPAMC: C, aldehyde cellulose (CNC-CHO); P, polyethylenimine (PEI); A, acrylic acid; M, 3-sulfonic acid propyl methyl acrylic acid potassium (MASEP); C, caffeic acid (CA). PCA: P, polyethylene glycol diacrylate (PEGDA); C, carboxylated cellulose (CNC-COOH); A, acrylic acid.

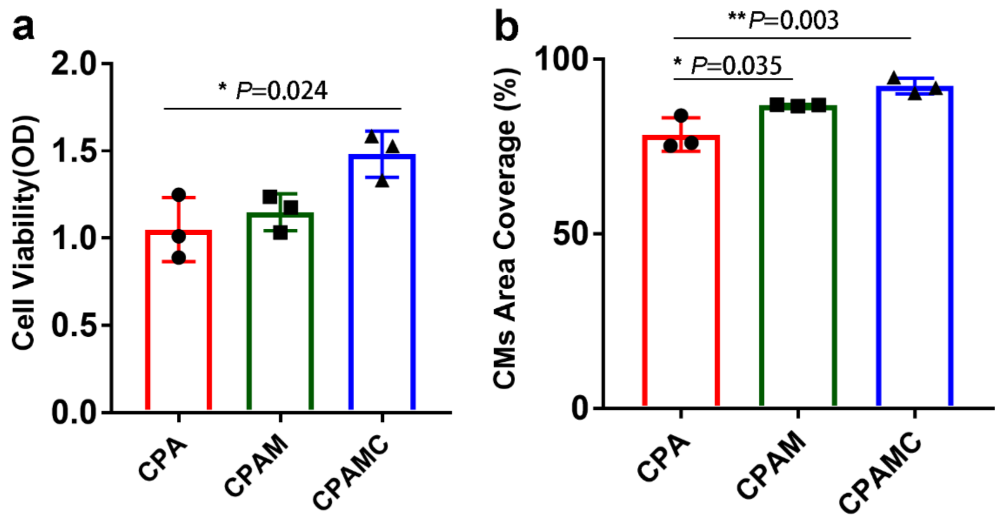

Supplementary Fig. 8. a) The CCK-8 results of CPA, CPAM and CPAMC hydrogels (n=3 independent samples). b) Quantitative CMs area percentage based on the live-dead staining of CPA, CPAM and CPAMC hydrogels (n=3 independent samples). Error bar means the standard deviation, *p < 0.05, **p < 0.01, ***p < 0.001, p value was generated by one-way analysis of variance (ANOVA), followed by Tukey's multiple-comparison post hoc test. n= independent samples. CPA, CPAM, CPAMC: C, aldehyde cellulose (CNC-CHO); P, polyethylenimine (PEI); A, acrylic acid; M, 3-sulfonic acid propyl methyl acrylic acid potassium (MASEP); C, caffeic acid (CA).

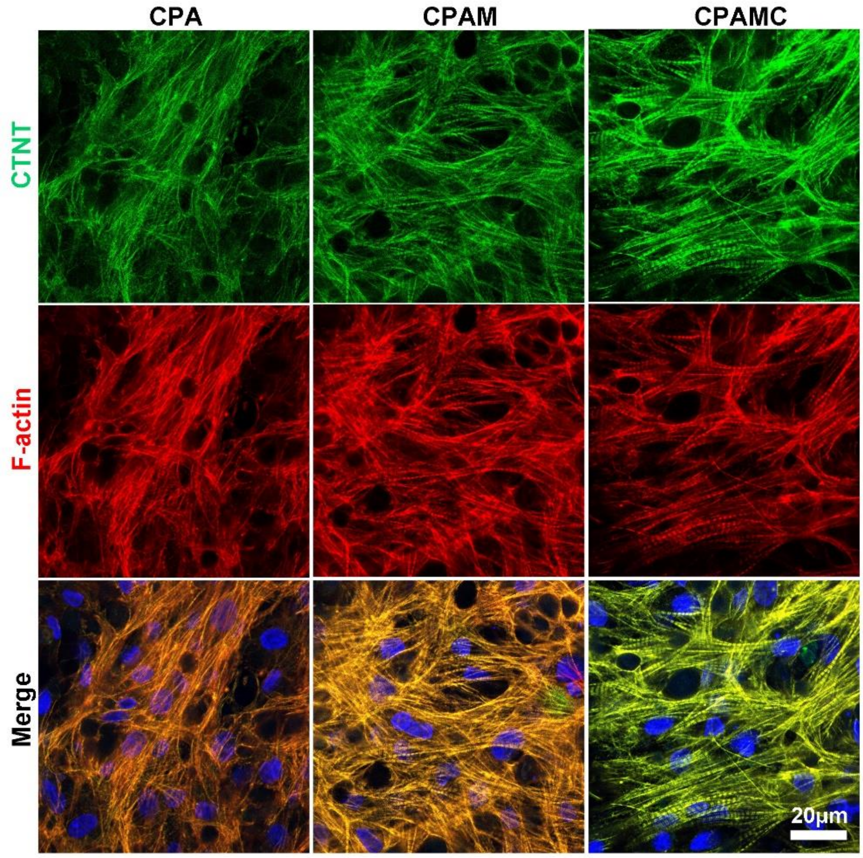

Supplementary Fig. 9. The F-actin staining and CTNT immunofluorescence staining of the CPA, CPAM and CPAMC hydrogels. CPA, CPAM, CPAMC: C, aldehyde cellulose (CNC-CHO); P, polyethylenimine (PEI); A, acrylic acid; M, 3-sulfonic acid propyl methyl acrylic acid potassium (MASEP); C, caffeic acid (CA).

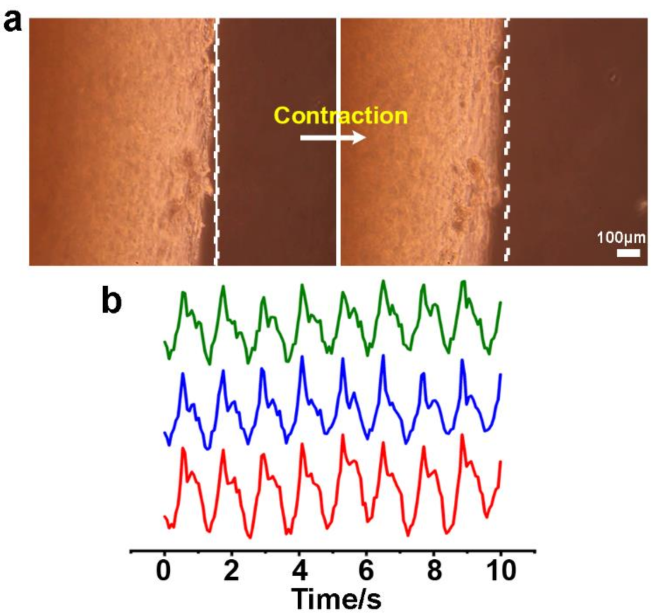

Supplementary Fig. 10. a) Photographs of CPAMC hydrogel cultured for 6 days displayed contraction behavior under a microscope field. b) Beating signal graphs of CPAMC hydrogel. CPAMC: C, aldehyde cellulose (CNC-CHO); P, polyethylenimine (PEI); A, acrylic acid; M, 3-sulfonic acid propyl methyl acrylic acid potassium (MASEP); C, caffeic acid (CA).

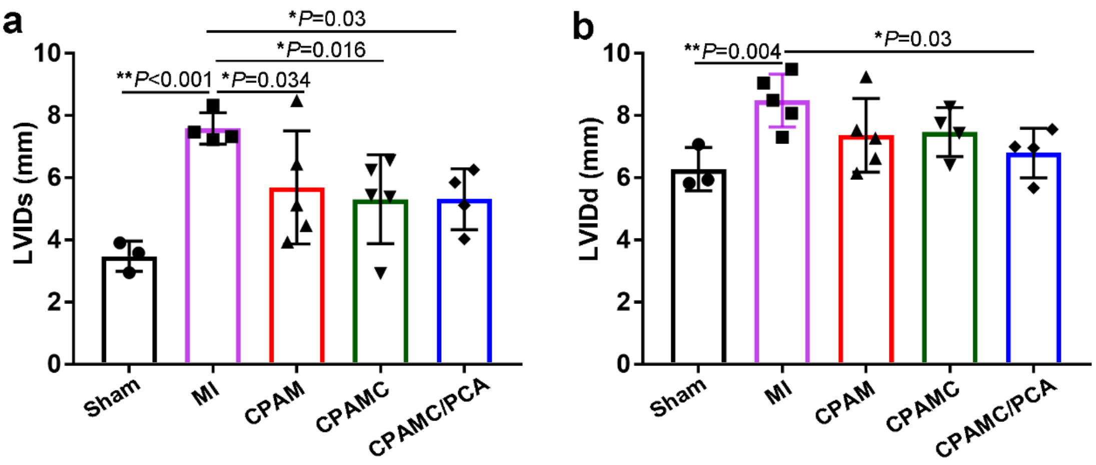

Supplementary Fig. 11. Typical echocardiographic parameters of the LVIDs (a) and LVIDd (b) for different groups (Error bar means the standard deviation, *p < 0.05, **p < 0.01, ***p < 0.001, p value was generated by one-way analysis of variance (ANOVA), followed by Tukey's multiple-comparison post hoc test, n= animals: Sham group, n=3; MI group, n=5; CPAM group, n=5; CPAMC group, n=5; CPAMC/PCA group, n=4).

CPAM, CPAMC: C, aldehyde cellulose (CNC-CHO); P, polyethylenimine (PEI); A, acrylic acid; M, 3-sulfonic acid propyl methyl acrylic acid potassium (MASEP); C, caffeic acid (CA). PCA: P, polyethylene glycol diacrylate (PEGDA); C, carboxylated cellulose (CNC-COOH); A, acrylic acid.

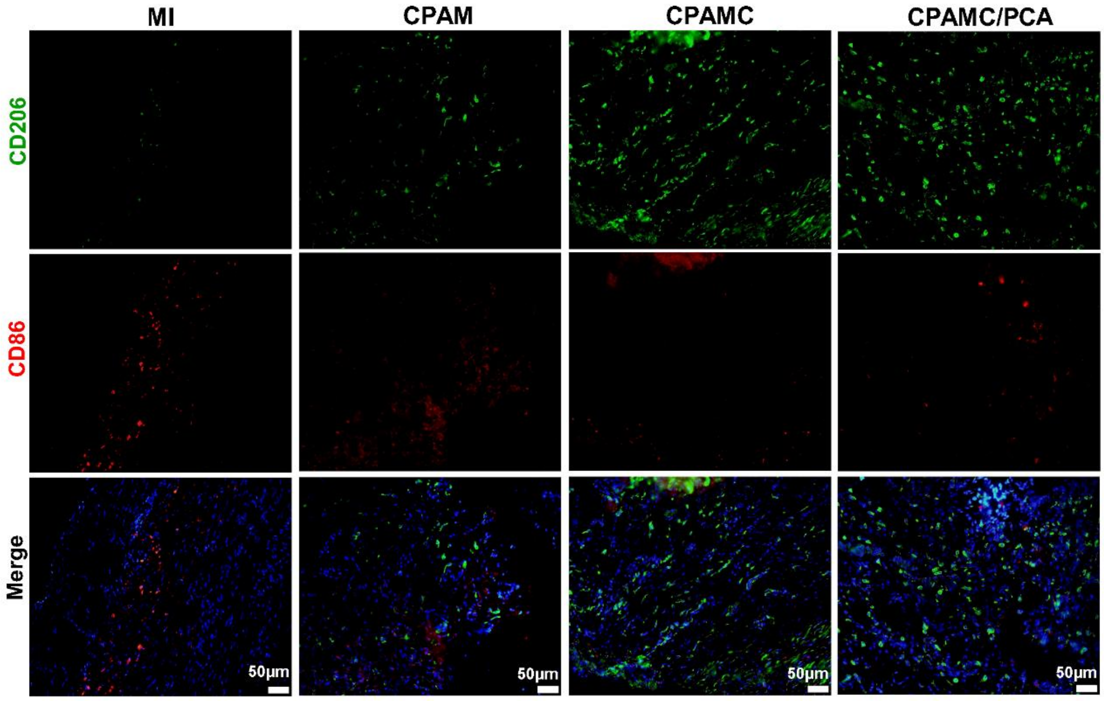

Supplementary Fig. 12. Representative CD86 (M1 macrophage marker, red) and CD206 (M2 macrophage markers, green) staining images of heart sections after *in-vivo* transplantation for 28 days. CPAM, CPAMC: C, aldehyde cellulose (CNC-CHO); P, polyethylenimine (PEI); A, acrylic acid; M, 3-sulfonic acid propyl methyl acrylic acid potassium (MASEP); C, caffeic acid (CA). PCA: P, polyethylene glycol diacrylate (PEGDA); C, carboxylated cellulose (CNC-COOH); A, acrylic acid.

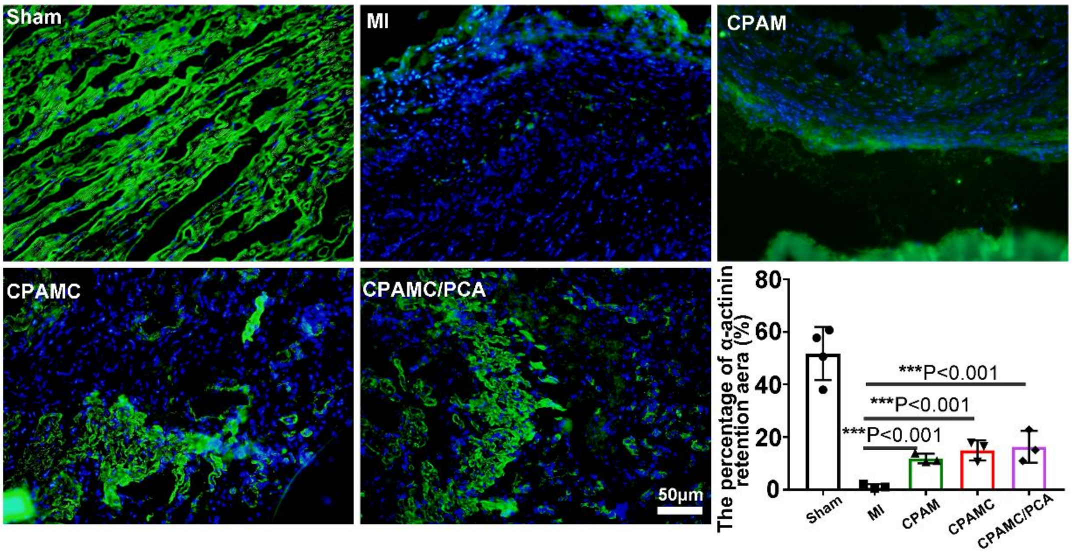

Supplementary Fig. 13. Cardiac-specific marker α-actinin (green) expression in the infarct region and patch region of the heart in different groups via immunostaining and their statistical analysis results (Error bar means the standard deviation, *p < 0.05, **p < 0.01, ***p < 0.001, p value was generated by one-way analysis of variance (ANOVA), followed by Tukey's multiple-comparison post hoc test, n= independent samples: Sham group, n=4; MI group, n=3; CPAM group, n=3; CPAMC group, n=3; CPAMC/PCA group, n=3). CPAM, CPAMC: C, aldehyde cellulose (CNC-CHO); P, polyethylenimine (PEI); A, acrylic acid; M, 3-sulfonic acid propyl methyl acrylic acid potassium (MASEP); C, caffeic acid (CA). PCA: P, polyethylene glycol diacrylate (PEGDA); C, carboxylated cellulose (CNC-COOH); A, acrylic acid.

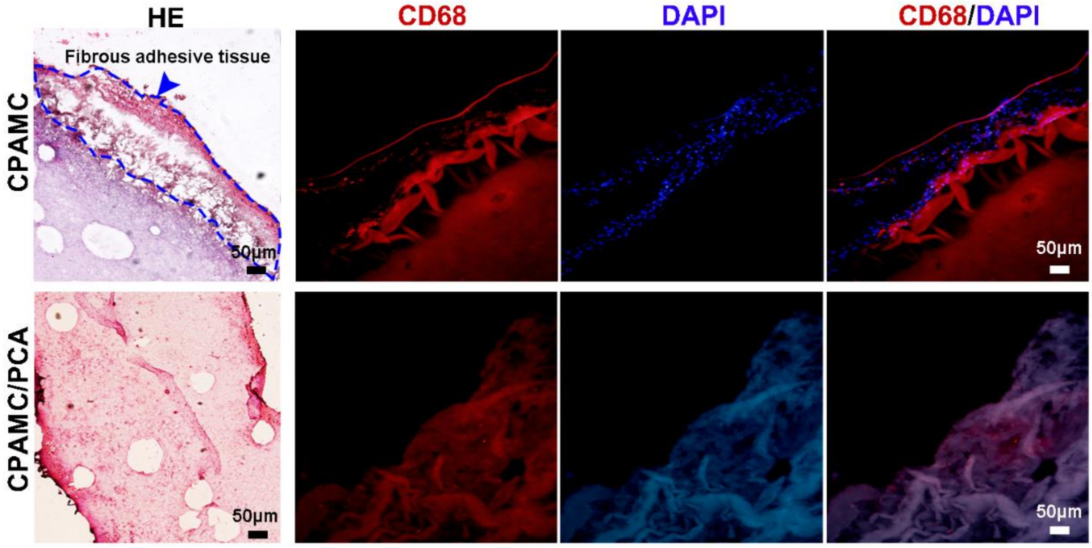

Supplementary Fig. 14. HE staining and immunofluorescence (CD68) were used to evaluate the inflammatory status of CPAMC ECP and CPAMC/PCA ECP. CPAMC: C, aldehyde cellulose (CNC-CHO); P, polyethylenimine (PEI); A, acrylic acid; M, 3-sulfonic acid propyl methyl acrylic acid potassium (MASEP); C, caffeic acid (CA). PCA: P, polyethylene glycol diacrylate (PEGDA); C, carboxylated cellulose (CNC-COOH); A, acrylic acid.
