# Supplementary material for: A smart adhesive Janus hydrogel for non-invasive cardiac repair and tissue adhesion prevention
Source: Nat Commun. 2022 Dec 12;13:7666. doi: 10.1038/s41467-022-35437-5 (PMC9744843; doi:10.1038/s41467-022-35437-5)
Supplement: Supplementary file 3 — Description of Additional Supplementary Files [file 41467_2022_35437_MOESM3_ESM.pdf]

Description of Additional Supplementary Files

Supplementary Movie 1: The remarkable adhesive strength of CPAMC hydrogel to heart tissue was shown to endure water flow.

Supplementary Movie 2: Instant adhesion of the CPAMC hydrogel to PP substrate underwater and an ability of the glued substrate to lift a weight of 50 g.

Supplementary Movie 3: The glued substrate of CPAMC hydrogel could lift a weight of 1.5 Kg after contact for 48 h at RT.

Supplementary Movie 4: GSH (30 wt.%) triggered facile detachment of the CPAMC hydrogel after treatment 5min.

Supplementary Movie 5: Comparison in the adhesion of the CPAMC side and PCA side of the CPAMC/PCA Janus hydrogel in binding the pig skin tissue.

Supplementary Movie 6: The calcium transient of CMs on different hydrogels at day 7 of culture.

Supplementary Movie 7: The spontaneous contraction activity of CMs seeded on CPAMC hydrogel culture different times.
