# Supplementary material for: A smart adhesive Janus hydrogel for non-invasive cardiac repair and tissue adhesion prevention
Source: Nat Commun. 2022 Dec 12;13:7666. doi: 10.1038/s41467-022-35437-5 (PMC9744843; doi:10.1038/s41467-022-35437-5)
Supplement: Supplementary file 11 — Reporting Summary [file 41467_2022_35437_MOESM11_ESM.pdf]

nature portfolio

Corresponding author(s): Honghao Hou and Xiaozhong Qiu

Last updated by author(s): Nov 19, 2022

# Reporting Summary

Nature Portfolio wishes to improve the reproducibility of the work that we publish. This form provides structure for consistency and transparency in reporting. For further information on Nature Portfolio policies, see our Editorial Policies and the Editorial Policy Checklist.

## Statistics

For all statistical analyses, confirm that the following items are present in the figure legend, table legend, main text, or Methods section.

| n/a | Confirmed | |
|---|---|---|
| ☐ | ☒ | The exact sample size (*n*) for each experimental group/condition, given as a discrete number and unit of measurement |
| ☐ | ☒ | A statement on whether measurements were taken from distinct samples or whether the same sample was measured repeatedly |
| ☐ | ☒ | The statistical test(s) used AND whether they are one- or two-sided<br>*Only common tests should be described solely by name; describe more complex techniques in the Methods section.* |
| ☒ | ☐ | A description of all covariates tested |
| ☒ | ☐ | A description of any assumptions or corrections, such as tests of normality and adjustment for multiple comparisons |
| ☐ | ☒ | A full description of the statistical parameters including central tendency (e.g. means) or other basic estimates (e.g. regression coefficient) AND variation (e.g. standard deviation) or associated estimates of uncertainty (e.g. confidence intervals) |
| ☐ | ☒ | For null hypothesis testing, the test statistic (e.g. *F*, *t*, *r*) with confidence intervals, effect sizes, degrees of freedom and *P* value noted<br>*Give P values as exact values whenever suitable.* |
| ☒ | ☐ | For Bayesian analysis, information on the choice of priors and Markov chain Monte Carlo settings |
| ☒ | ☐ | For hierarchical and complex designs, identification of the appropriate level for tests and full reporting of outcomes |
| ☒ | ☐ | Estimates of effect sizes (e.g. Cohen's *d*, Pearson's *r*), indicating how they were calculated |

*Our web collection on statistics for biologists contains articles on many of the points above.*

## Software and code

Policy information about availability of computer code

| | |
|---|---|
| Data collection | Zeiss Zen software (ver. 3.2) and Olympus BX53 software were used to acquire images |
| Data analysis | All statistical analyses have been conducted with the version20 of the IBM SPSS Statistics software, Image J, Origin 8 software and GraphPad Prism7. |

For manuscripts utilizing custom algorithms or software that are central to the research but not yet described in published literature, software must be made available to editors and reviewers. We strongly encourage code deposition in a community repository (e.g. GitHub). See the Nature Portfolio guidelines for submitting code & software for further information.

## Data

Policy information about availability of data

All manuscripts must include a data availability statement. This statement should provide the following information, where applicable:

- Accession codes, unique identifiers, or web links for publicly available datasets
- A description of any restrictions on data availability
- For clinical datasets or third party data, please ensure that the statement adheres to our policy

All study data are included in the article and/or Supporting Information. Source data are provided with this manuscript.

## Human research participants

Policy information about studies involving human research participants and Sex and Gender in Research.

| | |
|---|---|
| Reporting on sex and gender | The work did not involve human research. |
| Population characteristics | The work did not involve human research. |
| Recruitment | The work did not involve human research. |
| Ethics oversight | The work did not involve human research. |

Note that full information on the approval of the study protocol must also be provided in the manuscript.

# Field-specific reporting

Please select the one below that is the best fit for your research. If you are not sure, read the appropriate sections before making your selection.

☒ Life sciences ☐ Behavioural & social sciences ☐ Ecological, evolutionary & environmental sciences

For a reference copy of the document with all sections, see nature.com/documents/nr-reporting-summary-flat.pdf

# Life sciences study design

All studies must disclose on these points even when the disclosure is negative.

| | |
|---|---|
| Sample size | A minimum of 3 sample replicates (note that in each replicate sample) were performed. N is specified in figure legends. |
| Data exclusions | No data was excluded for this study. |
| Replication | The sample size in cell and animal experimental groups were ≥ 3 to ensure replicability. From the measured results, the data showed high similarity within the same testing group, and the replicability was good in each group. |
| Randomization | Rats were randomly allocated to treatment groups. Image acquisition was randomized for immunofluorescence. |
| Blinding | No blinding measures were deliberately taken during data collection and analysis, and all the data were processed by multiple authors. |

# Reporting for specific materials, systems and methods

We require information from authors about some types of materials, experimental systems and methods used in many studies. Here, indicate whether each material, system or method listed is relevant to your study. If you are not sure if a list item applies to your research, read the appropriate section before selecting a response.

### Materials & experimental systems

| n/a | Involved in the study | |
|---|---|---|
| ☐ | ☒ | Antibodies |
| ☐ | ☒ | Eukaryotic cell lines |
| ☒ | ☐ | Palaeontology and archaeology |
| ☐ | ☒ | Animals and other organisms |
| ☒ | ☐ | Clinical data |
| ☒ | ☐ | Dual use research of concern |

### Methods

| n/a | Involved in the study | |
|---|---|---|
| ☒ | ☐ | ChIP-seq |
| ☒ | ☐ | Flow cytometry |
| ☒ | ☐ | MRI-based neuroimaging |

## Antibodies

| | |
|---|---|
| Antibodies used | Anti-Sarcomeric Alpha Actinin (Mouse, ab9465, Abcam, 1:250); Anti-vWF (Rabbit, ab6994, Abcam, 1:250); Anti-CTNT (Mouse, ab10214, Abcam1:250); Anti-CX43(Rabbit, ab11370, Abcam1:1000); Anti-CD86(Mouse, ab220188, Abcam,1:200); Anti-CD206(Rabbit, ab64693, Abcam,1:200); Anti-α-SMA (Mouse, BM0002,1:200); Anti-TGF-β (Rabbit, ab31013, Abcam,1:250) Anti-iNOS (Mouse, ab49999, Abcam,1:250); Anti-CD68 (Mouse. ab955, Abcam, 1:200) Donkey anti-Rabbit IgG H&L (Alexa Fluor 568) (A10042, Invitrogen, 1:500), Donkey anti-Rabbit IgG H&L (Alexa Fluor 488) (A21206, Invitrogen, 1:500), Donkey anti-Mouse IgG H&L (Alexa Fluor 488) (A21202, Invitrogen, 1:500), Donkey anti-Mouse IgG H&L (Alexa Fluor 568) (A10037, Invitrogen, 1:500). |

| | |
|---|---|
| Validation | Mouse monoclonal [EA-53] to Sarcomeric Alpha Actinin (ab9465, Abcam, 1:250) Application: WB, IP, IHC-P, ICC/IF; Species Reactivity: Rat, Human.<br>Rabbit polyclonal to Von Willebrand Factor (Rabbit, ab6994, Abcam, 1:250) Application: WB, ICC/IF, IHC-Fr, Flow Cyt, IHC-P, IHC-FoFr, IHC-FrFl; Species Reactivity: Rat, Sheep, Horse, Guinea pig, Cow, Dog, Human, Pig.<br>Mouse monoclonal [1F11] to Cardiac Troponin T (Anti-CTNT) (ab10214, Abcam, 1:250) Application: ICC/IF, ELISA; Species Reactivity: Human, Recombinant fragment.<br>Rabbit polyclonal to Connexin 43 / GJA1 - Intercellular Junction Marker (rabbit,ab11370, Abcam, 1:1000) Application: IHC-Fr, ICC, IHC-P, WB; Application: Mouse, Rat, Hamster, Cow, Dog, Human, Pig, Monkey.<br>Mouse monoclonal [C86/1146] to CD86 (Mouse, ab220188, Abcam,1:200) Application: ICC, IHC-P, WB; Species Reactivity: Human.<br>Rabbit polyclonal to Mannose Receptor Anti-CD206 (Rabbit, ab64693, Abcam, 1:200) Application: IHC-P, WB, ICC; Species Reactivity: Mouse, Rat, Human.<br>Mouse monoclonal to Anti-α-SMA (Mouse, BM0002, Boster,1:200) Application: WB, IHC, IF; Species Reactivity: Mouse, Rat, Human.<br>Rabbit polyclonal to TGF-β (Rabbit, Abcam, 1:250) Application: ICC/IF, IP, IHC-P, IHC-Fr, WB; Species Reactivity: Mouse, Rat, Cow, Human.<br>Mouse monoclona to CD68 (Mouse. ab955 Abcam, 1:200) Application: WB,ICC.HC-P: Species Reactivitv: Human<br>Mouse monoclonal [NOS-IN] to iNOS (Mouse, ab49999, Abcam, 1:250), Application: ICC/IF, WB, Species Reactivity: Mouse.<br>Donkey anti-Rabbit IgG H&L (Alexa Fluor 568) (A10042, Invitrogen, 1:500), Application: ICC/IF; Species Reactivity: Rat.<br>Donkey anti-Mouse IgG H&L (Alexa Fluor 488) (A21202, Invitrogen, 1:500), Application: ICC/IF; Species Reactivity: Mouse. |

## Eukaryotic cell lines

Policy information about cell lines and Sex and Gender in Research

| | |
|---|---|
| Cell line source(s) | Neonatal rat cardiomyocytes (NRCMs) were isolated in our lab according to Wang, L., et al. Nat. Biomed. Eng. 5, 1157 (2021).<br>RAW264.7 cells was purchased from Cell Bank, Chinese Academy of Sciences, China |
| Authentication | No authentication as such was performed. However, at least three biological replicates were preformed for each donor cells to confirm the consistency of the cell lines |
| Mycoplasma contamination | I confirmed that NRCMs and RAW264.7 cells tested negative for mycoplasma contamination. |
| Commonly misidentified lines<br>(See ICLAC register) | There were no misidentified cells in our studies. |

## Animals and other research organisms

Policy information about studies involving animals; ARRIVE guidelines recommended for reporting animal research, and Sex and Gender in Research

| | |
|---|---|
| Laboratory animals | 8 weeks old male SD-rats were purchased from the Animal Institute of Southern Medical University (Guangzhou, China). |
| Wild animals | This study did not involve wild animals |
| Reporting on sex | Male |
| Field-collected samples | This study did not involve samples collected from the field. |
| Ethics oversight | All animal procedures were approved by the welfare and Ethical Committee for Experimental Animal Care of Southern Medical University. |

Note that full information on the approval of the study protocol must also be provided in the manuscript.
